# An Investigation of the Relationship Between Isolated Anti-dense Fine-Speckled 70 Autoantibodies and Allergen-Specific Immunoglobulin E

**DOI:** 10.7759/cureus.38494

**Published:** 2023-05-03

**Authors:** Alper Togay, Nisel Yılmaz

**Affiliations:** 1 Medical Microbiology, Health Science University İzmir Tepecik Training and Research Hospital, İzmir, TUR

**Keywords:** allergen-specific ige, allergic patients, autoimmune disease, autoallergy, anti-dfs70

## Abstract

Background

Although the mechanisms of the formation of anti-dense fine-speckled 70 (anti-DFS70) antibodies are not fully known, there is evidence in the literature that allergic reactions may play a role in their formation. Immunoglobulin E (IgE)-mediated immunopathological mechanisms are increasingly being elucidated in diseases such as atopic dermatitis and urticaria-related diseases. We aimed to reveal its relationship with anti-DFS70 in allergen-sensitive patients with positive specific IgE (sIgE) levels.

Methodology

The study included samples of 758 patients who underwent antinuclear antibody (ANA) screening and allergen-sIgE testing between January 2019 and January 2022. Patients’ clinical diagnoses were retrospectively obtained from the hospital information management system. ANA was tested according to the instructions of the manufacturer by the indirect immunofluorescent antibody method using HEp-2 cell substrates (Euroimmun Luebeck, Germany). Allergen-sIgE was determined by chemiluminescence on the Immulite 2000 XPI system (Siemens Healthcare Diagnostics Products GmbH, Marburg, Germany) according to the instructions of the manufacturer.

Results

ANA pattern was detected in 74 samples included in the study. ANA-positive patients were divided into DFS70 (+) and DFS70 (-) groups. A statistically significant increase in the DFS70 pattern was observed in patients with a positive allergen-sIgE test (p < 0.0001). Both allergen-sIgE and DFS70 positivity were statistically significant in younger age groups (p < 0.05). The most common diagnosis was urticaria-related conditions in 23 (31%) patients with a positive allergy test.

Conclusions

Our study shows that the positivity of the DFS70 pattern is increased in allergen-sensitive patients. Therefore, the allergen-sIgE-mediated allergic disease should be considered in patients with isolated anti-DSF70. Studies with related disease groups are needed to determine whether there is a relationship between anti-DFS70 and allergy-related disease in these patients. If an immunopathological mechanism is not found, these false-positive results can be considered clinically insignificant, and unnecessary consultations can be avoided.

## Introduction

Autoantibodies detected in systemic autoimmune rheumatic diseases are usually directed against nuclear antigens and are called antinuclear antibodies (ANAs). Diseases in which ANAs are positive belong to a broad spectrum of diseases and are referred to as ANA-related rheumatic diseases (AARDs) [1]. The indirect immunofluorescent antibody (IIF) method is the gold standard for screening ANA [2]. With this method, not all ANAs are associated with AARDs. The dense fine-speckled 70 (DFS70) pattern is one of the most common patterns when scanning with IIF [3]. This pattern shows staining in metaphase and interphase cell nuclei, where granules typically differ in size and gloss [4].

DFS70 antibodies are commonly found in the serum of healthy individuals [5-7]. Isolated anti-DFS70 antibody positivity is found in less than 5.7% of systemic rheumatic diseases [8]. Vogt-Koyanagi-Harada disease is an inflammatory multisystem autoimmune disease with ocular, auditory, skin, and neurological involvement and is the most common condition for anti-DFS70 antibody positivity (66.7%) [9]. Less frequently, it is observed in non-systemic autoimmune and allergic diseases such as atopic dermatitis (AD), interstitial cystitis, asthma, chronic inflammatory diseases, and cancers [10].

On the other hand, immunoglobulin (Ig) E plays a well-documented role in the classical allergy pathway to exogenous allergens. The importance and cellular and molecular implications of IgE autoantibodies in various diseases are still being researched, but the link between IgE antibodies and autoimmunity is increasing [11]. ﻿Watanabe et al. [12] identified not only IgG but also IgE autoantibodies against DFS70. IgG antibodies to DFS70 have been found in patients with AD, particularly in patients with facial involvement [13]. More recently, IgE-mediated autoallergy has been demonstrated in other diseases such as chronic spontaneous urticaria (CSU) [14,15] and chronic inducible urticaria (CindU) [16].

Environmental stressors, including allergens, can trigger oxidative events and inflammation in certain tissues, resulting in a response characterized by an increase in the DFS70 antigen. There is evidence that overexpression of intracellular autoantigens in a pro-inflammatory environment can lead to the formation of autoantibodies [17]. Therefore, it may be helpful to determine why anti-DFS70 develops in allergen-sensitive patients. This study aimed to investigate the anti-DFS70 relationship in patients with specific IgE (sIgE)-positive allergic symptoms and provide helpful data for the diagnosis of etiology in patients with anti-DFS70 positivity.

## Materials and methods

Study population

Among 28,506 serum samples submitted from different clinics for ANA screening, 7,382 specific allergy tests were examined in the Department of Medical Microbiology Laboratory of Health Science University İzmir Tepecik Training and Research Hospital between January 2019 and January 2022. Our study group comprised 758 patients being tested for both ANA and allergen-sIgE. Patients’ clinical diagnoses were obtained retrospectively from medical records.

ANA screening

ANA was tested according to manufacturer instructions by the IIF method using HEp-2 cell substrates (Euroimmun Luebeck, Germany). IIF method was screening dilution 1:100. After slide preparation, the two experts identified the DFS patterns as the anticellular 2 patterns according to the International Consensus on ANA models [18].

The DFS staining pattern is defined as dense fine-speckled staining of the nucleoplasm of HEp-2 cells and the chromosomal plate of metaphase cells with heterogeneity in size, brightness, and density of speckles. All IIF-ANA-positive sera were classified into either a DFS70 (+) group or a DFS70 (-) group according to the IIF-ANA pattern.

Allergen-sIgE detection

Allergen-sIgE was determined by chemiluminescence on the Immulite 2000 XPI system (Siemens Healthcare Diagnostics Products GmbH, Marburg, Germany) according to the manufacturer’s instructions. Values below 0.35 IU/mL were considered negative. Values of 0.35 IU/mL and above were divided into six classes (I to VI), and the results were reported according to the manufacturer’s instructions. Class I was between 0.35 and 0.69 IU/mL; class II was between 0.7 and 3.4 IU/mL; class III was between 3.5 and 17.4 IU/mL; class IV was between 17.5 and 49.9 IU/mL; class V was between 50 and 100 IU/mL; and class VI was above 100 IU/mL. Patients were tested for the following different panels: food panel 1 (coconut, peanut, Brazil nut, hazelnut, almond); food panel 5 (soybean, codfish, milk, wheat, peanut, egg white); tree panel 7 (melaleuca, willow, eucalyptus, acacia, white pine, olive); inhalant panel 8 (birch, cat dander-epithelium, timothy grass, dog dander, *Cladosporium herbarum,* cultivated ryegrass, *Dermatophagoides pteronyssinus*, mugwort); dust panel 1 (cockroach, *D. farinae*, house dust - Greer, *D. pteronyssinus*); mite panel 1 (*Euroglyphus maynei*, *D. microceras, Lepidoglyphus destructor, Tyrophagus putrescentiae, D. farina, D. pteronyssinus, Glycyphagus domesticus, Blomia tropicalis*); animal panel 2 (rat, dog dander, guinea pig epithelium, cat dander epithelium, mouse); and grass panel 1 (June grass, perennial ryegrass, meadow fescue, timothy grass, orchard grass, foods, trees, inhalants, dust, mites, animals, and grasses) to determine their allergy status.

Ethical approval

This study was performed after obtaining approval of the Ethics Committee of Health Science University İzmir Tepecik Training and Research Hospital (approval number: 2021/11-17; date: 11/15/2021) ﻿and performed in accordance with the ethical standards laid down in the 1964 Declaration of Helsinki.

Statistical analysis

The chi-square test was used to compare the categorical variables of allergy test positivity, ANA test positivity, ANA test dilution, and sex. Comparisons between the levels of the categorical variables in relation to the age of the patients were performed using the Mann-Whitney U test and the Kruskal-Wallis test. Statistical analyses were performed using SPSS statistics version 21 (IBM Corp., Armonk, NY USA). A p-value <0.05 was considered statistically significant. Data are presented as mean ± standard deviation (SD), number (n), and percentage (%).

## Results

**Figure 1:**
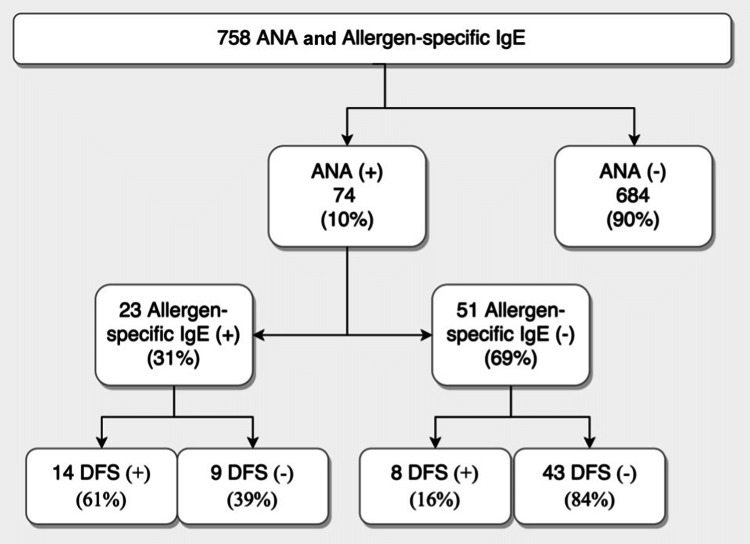
Flowchart of the study population. ANA: antinuclear antibody; DFS70: dense fine-speckled 70; IgE: immunoglobulin E

The selected cohort included 758 sera (Male = 252, Female = 506) from children and adults analyzed for routine testing for ANA and allergen-sIgE (Figure 1). The selected cohort’s mean age was 27.44 ± 19.13 years (the mean age of females was 30.28 ± 17.95 years, and the mean age of males was 22.63 ± 20.77 years). ANA pattern was detected in 74 samples included in this study. ANA-positive results were divided into DFS70 (+) and DFS70 (-) groups. There was a statistically significant dependence between ANA test results and allergy test results (p < 0.0001) (Figure 1). There was no correlation between IIF test dilution and allergy test classes.

**Figure 2:**
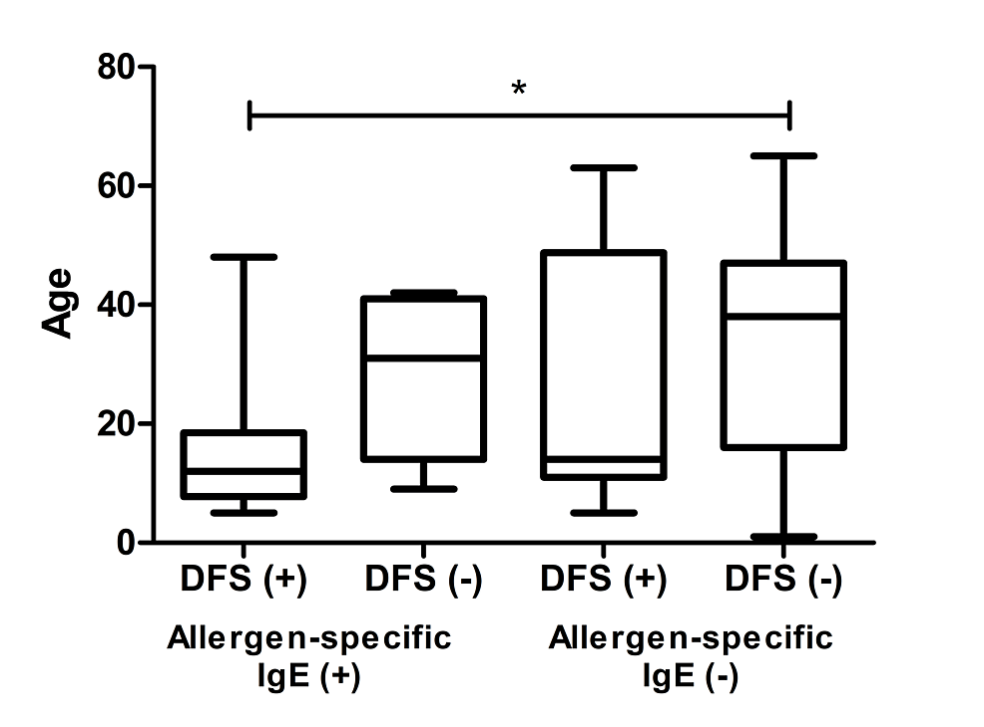
Age distribution of the ANA(+) population (n = 74). ANA: antinuclear antibody; DFS70: dense fine-speckled 70; IgE: immunoglobulin E; *: p<0.05

There was a statistically significant dependence between study groups and age (p = 0.02, Kruskal-Wallis test). The Dunn test was applied for further pairwise analysis. A significant difference was observed only between the ages of DFS (+)-allergen sIgE (+) and DFS (-)-allergen sIgE (-) (p < 0.05) (Figure 2). A total of 22 DFS70 (+) and 52 DFS70 (-) samples were identified in the selected cohort.

The mean age values among DFS70 (+) and DFS70 (-) groups were statistically significantly different (p = 0.005, Mann-Whitney U test). The DFS70 (+) group was significantly younger compared to the individuals in the DFS70 (-) group. There was no significant difference between the sex of the DFS70 (+) and the DFS70 (-) group (p = 0.144). A total of 23 allergen-sIgE-positive and 51 allergen-sIgE-negative samples were identified in the selected cohort. See Table 1 for details on the distribution of age and sex of the ANA-positive group and the allergen-sIgE group.

**Table 1:** The distribution of age and sex of the ANA (+) population (n = 74). ANA: antinuclear antibody; DFS70: dense fine-speckled 70; SD: standard deviation; sIgE: specific immunoglobulin E

	Age, mean ± SD (years)	Women, n (%)
DFS70 (+) group (n = 22)	18.55 ± 16.45	13 (59)
DFS70 (-) group (n = 52)	33.14 ± 19.13	41 (79)
P-values	0.005	0.144
Allergen-sIgE positive (n = 23)	20.00 ± 13.59	14 (61)
Allergen-sIgE negative (n = 51)	32.77 ± 19.53	40 (78)
P-values	0.015	0.196

The mean age was 28.8 ± 18.77 years (range = 1-65 years) in 74 patients, and there was no normal distribution with respect to age. The sex distribution of the subjects included in our study was 73% (54/74) women and 27% men (20/74). The median age of women and men was statistically significant (p = 0.008, Mann-Whitney U test). The mean age of women was 32.44 ± 19.29 years (range = 1-65 years). The mean age of men was 18.95 ± 13.26 years (range = 2-43 years).

When specific IgE-positive patients were examined according to their clinics, urticaria-related conditions were the most common. There was no significant difference between the DFS70 (+) group with urticaria-related conditions (p = 0.417). See Table 2 for details on the characteristics of allergen-sIgE-positive patients.

**Table 2:** Characteristics of allergen-sIgE-positive patients (n = 23). ANA: antinuclear antibody; DFS70: dense fine-speckled 70; sIgE: specific immunoglobulin E; M: male; F: female; * +: ﻿>1/100-<1/320; ++: ≥1/320-<1/1,000; +++: ﻿≥1/1,000-<1/3,200; ++++: >1/3,200; Fp: food panel; Ip: inhalant panel; Gp: grass panel; Tp: tree panel; Mp: mite panel

	Sex	Age	Condition	Dilutions of ANA*	Class scores	Positive allergen panel
DFS70 (+) (n = 14)	M	5	Chronic urticaria	+	I	Fp 5
	F	6	Allergic rhinitis	+	I	Fp 5
	M	7	Acute urticaria	+	I	Ip 8
	M	8	Chronic urticaria	+	I	Fp 5
	F	9	Chronic urticaria	+++	I	Fp 1
	F	10	Acute urticaria	++	I	Fp 5
	M	12	Chronic urticaria	+	III	Ip 8
	F	12	Allergic rhinitis	++	IV	Ip 8
	M	13	Chronic urticaria	++	I	Ip 8
	F	17	Chronic urticaria	+	I	Ip 8
	M	18	Chronic urticaria	+++	III	Ip 8
	F	20	Allergic rhinitis	+	V	Gp 1
	F	26	Allergic rhinitis	++	II	Tp 7
	F	48	Allergic rhinitis	+	I	Ip 8
DFS70 (-) (n = 9)	F	9	Acute urticaria	+	II	Fp 5
	F	12	Chronic urticaria	+++	III	Ip 8
	F	16	Allergic rhinitis	++	V	Ip 8
	M	17	Allergic conjunctivitis	+	I	Ip 8
	F	31	Contact dermatitis	++	II	Ip 8
	F	40	Allergic rhinitis	+++	I	Mp 1
	M	41	Chronic urticaria	+	I	Ip 8
	M	41	Acute urticaria	++	II	Tp 7
	F	42	Allergic rhinitis	++	II	Ip 8

## Discussion

Environmental factors that increase oxidative stress and inflammation include infectious agents, unhealthy diet, irradiation, pro-oxidants, and allergens. DFS70 is thought to support cellular protection against environmental stressors [17]. There is evidence that overexpression of intracellular autoantigens in a proinflammatory context can trigger the formation of autoantibodies [17]. The presence of anti-DFS70 has also been associated with inflammatory diseases such as AD [12,13]. The immunopathology of diseases such as AD and CSU, which is thought to be an autoimmune disease closely related to allergic diseases, may be important in understanding why anti-DFS70 antibodies develop in patients who are sensitive to allergens. The results show that the DFS70 pattern is statistically significantly more prevalent in allergen-sensitive patients (p < 0.0001).

IgE autoreactivity was first discovered and described in AD, where IgE autoantibodies are very common [17,19]. IgE autoantibodies directed against DFS70 and a variety of autoantigens have been identified in AD [11,12]. Immunohistochemical findings associated with DFS70 were detected in skin sections of AD patients [12]. More recently, IgE-mediated autoallergy has been demonstrated in other disorders such as CSU [14,15] and CindU [16]. Urticaria-related conditions were the most common disease in our patient population. The prevalence of IgE anti-DFS70 autoantibodies in patients with urticaria symptoms was not determined, but Watanabe et al. [12] detected IgE-anti-DFS70 autoantibodies in 15% (nine of 61) of AD patients. Four of 61 AD patients were both IgE- and IgG-anti-DFS70 autoantibody-positive [12]. Therefore, it may be useful to determine the presence of IgE-type autoantibodies in addition to IgG anti-DFS70 autoantibodies with a larger population of patients with urticaria symptoms. Our patient population was not homogeneous. In addition to patients with urticarial symptoms, there were also allergic diseases. As reported in previous studies [10], we found high DFS70 in these patients with allergies. We believe that uncovering these mechanisms will be beneficial for the durable treatment of these patients. Or conversely, it could be argued that much of this cross-reactivity, while problematic for diagnostic analysis, is clinically insignificant as a false-positive result.

In our study, allergen-sIgE levels were higher at younger ages (p = 0.015). The study by Amici et al. [20] included 6,370 patients with allergic rhinitis and/or asthma with a mean age of 21.7 years and showed a discrepancy between changes in total and specific IgE levels with age. Allergen-sIgE levels typically decreased with age, but total IgE production did not decrease with age [20]. Our results are consistent with the findings of this study. The mean age of the specific IgE-positive group is 20.0 years.

The study by Albesa et al. [7], which analyzed the prevalence of anti-DFS70 antibodies in different geographic regions in the adult group, showed that antibodies were most common in young women. In our study, there was no significant difference between the sexes, but anti-DFS70 antibodies were also detected mainly in young women.

Our study had several limitations. First, our sample size was small. It is necessary to investigate a larger population of allergic or allergen-sensitized patients with different disease groups. Second, because this study was retrospective, we were unable to determine whether the anti-DFS70 antibodies produced in patients with IgE to an allergen were of the IgG-only type. Prospective studies can be designed by forming a larger study group than the disease groups that may be associated with this condition.

## Conclusions

Our data show an increased positivity of the DFS70 pattern in patients with positive allergen-sIgE which could be associated with the disease pathogenesis. Therefore, if ANA is requested and only the DSF70 pattern is detected, physicians may also consider allergen-specific sensitization or allergy, especially in younger patients. Further studies are needed to determine whether there is a relationship between anti-DFS70 and allergy-related diseases in these patients. If an immunopathological mechanism is not found, these false-positive results can be considered clinically insignificant, and unnecessary consultations can be avoided.
